# Extra-curricular supervised training at an academic hospital: is 200 hours the threshold for medical students to perform well in an emergency room?

**DOI:** 10.1186/1749-7922-7-S1-S12

**Published:** 2012-08-22

**Authors:** Phillipe Abreu-Reis, Guilherme Czelusniak Oliveira, Arthur Curtarelli de Oliveira, Hammad Sadique, Adonis Nasr, Flávio Daniel Saavedra Tomasich

**Affiliations:** 1 Federal University of Parana, Curitiba, Brazil; 2 Department of Surgery, Federal University of Parana, Curitiba, Brazil; 3 University Positivo, Curitiba, Brazil; 4 Hospital do Trabalhador, State Health Department, Parana, Brazil; 5 University of Birmingham, UK

## Abstract

**Introduction:**

Due to high number of jobs in Emergency Medicine (EM) and the lack of specialist to work in this field, recent graduates work in the emergency room straight after medical school. Additional courses on EM are available through Academic Leagues. This organizations offer lectures and supervised extra-curricular practical activities in their teaching university-affiliated hospital. The objectives of the present study are to assess the influence of hours undertaken in the extra-curricular practical activities on the performance and confidence of students in carrying out the different procedures in the emergency department, and on their own perception of how well they did. Also, to assess the influence the practical activities have on student´s future choice of specialty.

**Methods:**

A Cross-sectional study conducted by collecting data through a questionnaire. 102 eligible individuals were included and divided into two groups according to the number of extra-curricular hours performed (Group 1- up to 200 hours and Group 2- over 200 hours).

**Results:**

Students in Group 2 (over 200 hours) had a greater number of procedures performed on all variables evaluated, in particular, initial patient care (mean 363.8 vs.136.905 in Group 1 - p = 0.001), Simple Sutures (mean of 96.2 vs 33.980 respectively) ( p = 0.00003). To determine patient follow-up by the student, the number of initial patient care was correlated with number of discharge procedures performed (in Group 1, 49.6% of patients were not followed up and discharged by the same students who first talked to them in the hospital. While in Group 2, this value becomes 29.4 % - values for Group 1 - p = 0.011 and Group 2 - p = 0.117). Regarding the influence of the practical extra-curricular activities, 76.5% of the total reported that it had influenced their choice of future specialty.

**Conclusions:**

The aptitude, confidence and skill of students are closely linked to the practice time (number of training hours served). Two hundred hours appeared to be a relatively significant time for the student to demonstrate good conduct and ability. Practical extra-curricular activities had the ability to influence the future choice of specialty, either positively or negatively.

## Introduction

Emergency Medicine (EM) is a fascinating area to most medical students. However in developing countries like Brazil, EM is not considered a specialty. Consequently EM is taught to medical students as part of their core curriculum inside rotations, but no emergency dedicated residency training exists (like in other parts of the world), leaving gaps in medical training in these countries. Due to high number of jobs in EM and the lack of specialist to work in this field, recent graduates in developing countries sometimes work in the emergency room straight after medical school and have to either acquire knowledge by experience only or take additional courses during their graduation. Examples of additional courses in EM include the ATLS (Advanced Trauma Life Support), Trauma academic weeks, conferences and lectures. A more feasible alternative for countries like Brazil has been the formation of extra-curricular groups linked to both academic and non-academic hospitals where students are taught by qualified teachers, and thus complement their learning in specific areas such as EM [1-4]. In Brazil, these groups are known as "Academic leagues."

Academic Leagues offer lectures and supervised extra-curricular practical activities in their teaching university-affiliated hospital and form part of an overall parallel curriculum. The name Academic Leagues come from medical students creating these activities in order to acquire theoretic and practical experience [1,2]. This parallel curriculum has become essential for medical students in Brazil due to the gaps in Medical School core teaching and the amount of learning and training medical students need to be competent clinicians. Tavares et al. showed that 82.5% of medical students of a Brazilian University actively take part in the “Parallel curriculum”, spending on average 8.2 hours per week [2]. Furthermore, a similar study in the Brazilian state of Alagoas demonstrated that by the third year of medical school, 98.4% of the students are involved in some form of extracurricular activity [3] and for 12.5% of them, these activities lasted for more than 12 hours per week [3].

Extra-curricular activities in non-teaching hospitals without University affiliation may influence career choices as well. A study of medical students involved in extra-curricular activities in Critical Care Medicine in the city of Salvador, Brazil, concluded that the students’ in a career in Critical Care rose from 32% to 65% the establishment of an Academic League in this field [1]. Extra-curricular activities also boost good social work practice [3], providing valuable experience in dealing with death, suffering and feelings of powerlessness [4].

Some authors dispute the importance of the Academic Leagues in the training of medical students. Despite their potential benefits, these authors warn of the possible risk of premature specialization and too much practical work without being accompanied by theoretical knowledge, which can skew medical training [5].

The Hospital do Trabalhador in the city of Curitiba, Brazil, is a well-established Level I Trauma Center. It has the only emergency department in the city that utilizes an "open door system” (where the citizen can seek assistance directly) without referral by other hospitals or physicians. The Emergency Room of the Hospital do Trabalhador admitted 63,057 patients in 2010 and performed approximately 1,500 surgeries per month [6]. This public hospital is covered exclusively by the Brazilian Unified Health System (SUS). The hospital offers residency programs in general surgery and orthopedics/trauma. The hospital currently has 140 medical students in a supervised extra-curricular program. For this program, medical students are divided into teams that work according to a pre-established system of shifts. Medical students are selected for this extra-curricular program by examination. In order to be eligible for the exam, students must be in at least their fourth year and be regular students of one of the 4 medical schools in the region. The range of activities that can be undertaken in this extra-curricular program is broad and includes trauma, orthopedics and general surgery. The minimum number of hours required to complete the program is 250 hours (and the maximum allowed is 500 hours) over a maximum period of 12 months. The objective of this supervised program is to expose the student to everyday situations in trauma, teach how to diagnose and treat these diseases as well as help in decisions about their future specialty. The objectives of the present study are to assess the influence of hours undertaken in the extra-curricular practical activities on the performance and confidence of students in carrying out the different procedures in the emergency department, and on their own perception of how well they did. Also, we aim to assess the influence that the clerkship has on the student´s future choice of specialty.

## Methods

A Cross-sectional study conducted by collecting data through a questionnaire developed by the research group consisting of three parts. The first part recorded general information about the student i.e. name, semester, university, etc. The second part recorded an estimated number of procedures performed routinely in surgical emergency department. The student was also asked to evaluate themselves on how confident they were, how much their previous training contributed to their ability, how helpful supervision was (by residents and the attendings) and to record a score on a scale of 0-10 for each of these fields. The third part recorded how much the clerkship influenced their future career choice by closed (yes/no) questions.

The inclusion criteria of the study were all students who were studying medicine and participated in the surgical emergency medicine clerkship of the Hospital do Trabalhador in the second half of 2011.

The exclusion criteria of the study were all students who did not attend the annual meeting or who refused to complete the questionnaire.

If one (or more) of the three sections of the questionnaire had incomplete fields, that section(s) was removed but the remaining data was still included in the statistical analysis.

The students were divided into two groups: the first contained the students with less than 200 hours on duty in the emergency room and the second group contained those that had 200 hours or more on duty. Data was tabulated in spreadsheet format and analyzed using SPSS 19 software IBM. We used the non-paired non-parametric t-test.

Data was collected during the Annual General Meeting (AGM) of students at the Hospital do Trabalhador.

## Results

Of the 140 students in the emergency clerkship, 38 did not meet the inclusion criteria and were excluded, leading to a total of 102 questionnaires eligible for analysis. These 102 subjects were divided into two groups, Group 1 (with less than 200 hours of training in the clerkship = 71 students) and Group 2 (those with 200 or more hours of training in the clerkship = 31 student) and were analyzed against each other in each of the objectives mentioned above.

When comparing the number of patients: Group 2 students took care of an average 363.8 initial evaluations, whilst Group 1 students took care of an average of 136.9, giving an absolute difference of 226.9 patients, corresponding to an increase of 167.5 % by Group 2. The comparison between the two groups was statistically significant (p = 0.001075). (Table 1)

**Table 1:** Number of procedures versus number of hours of extra-curricular supervised activities.

Number of Procedures	< 200h	> 200h	p value
History Takings in Initial Patient Care	136.905	363.800	0.001075
Non-cast immobilizations	19.360	63.577	0.005303793
Cast immobilizations	32.811	102.160	0.000295235
Simple sutures	33.980	96.200	0.000032826369841
Donatti sutures	5.283	12.214	0.019836
Trauma Resuscitation Room visits	2.804	21.036	0.000045965

**Figure 1:**
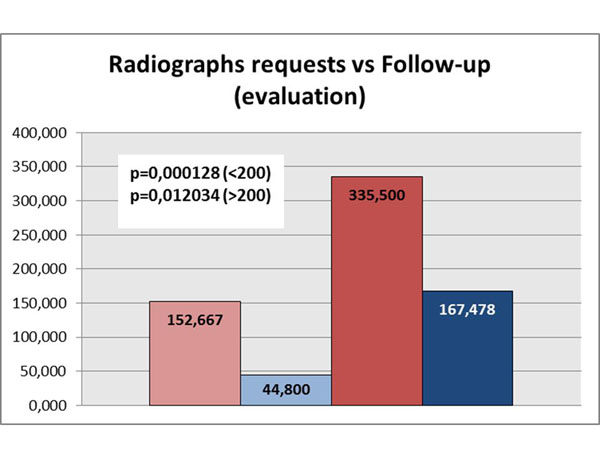
**Number of Radiographs Requests vs number of Radiographs evaluation/follow-up.** Rose: Group 1 requests. Light-Blue: Group 1 evaluations/follow-ups. Red: Group 2 requests. Dark-Blue: Group 2 evaluations/follow-ups.

Under the guidance and supervision of ED doctors, medical students have the ability to request imaging to those patients requiring further investigation. Students are then requested to follow-up the results afterwards, with the attending physician. Evaluation of the student radiographs revealed that Group 1 students requested an average of 152.6 radiographs while Group 2 students requested an average of 335.500 radiographs, an absolute increase of 182.8 examinations which represents a 119.7% increase for Group 2. When comparing the numbers of radiographs requested with the numbers of radiographs followed up with the attending/radiologist, Group 1 had an average of 44.8 radiographs followed up and Group 2 had an average of 167.4 followed up/evaluated (giving a difference of 122.6 – an increase of 273.8%). (p = 0.000128 for Group 1)( p = 0.012034 for Group 2). (Figure 1)

The results comparing the orthopedic immobilization procedures were divided into plastered and non-plastered. Regarding non-plaster immobilizations, it was observed that students in the Group 1 on average performed 19.3 procedures and Group 2 students performed on average 63.5 procedures (resulting in an increase of 44.2 immobilizations for Group 2, that represents a 229% increase – p value = 0.0053). In addition to this, for plaster immobilizations, Group 1 students on average performed 32.8 procedures compared to an average of 102.1 procedures by Group 2. This represents an increase of 69.3 procedures (211.2% more plaster immobilization) for Group 2 (p = 0, 00029). (Table 1)

We also collected data of surgical procedures such as sutures of skin lacerations, with a simple separated or a Donatti stitch. Once again, under supervision of ED doctors, students are able to perform these procedures. Group 1 students averaged 33.9 single stitch sutures, while Group 2 students averaged 96.2 of the same procedure (a difference of 183.7% of procedures, p = 0.000032). Regarding Donatti stitches, Group 1 students reported having done an average of 5.2 sutures, while in Group 2 the recorded average was 12.2, with a difference of 7 procedures (131% more for Group 2). (Table 1)

Students have an established role in the Emergency Department, but sometimes their help is needed for trauma patients in the resuscitation room. The student on duty estimated the number of supervised visits to the trauma resuscitation room. Group 1 showed a mean of 2.8 visits, compared with a mean of 21 visits in Group 2 (an increase of 650.2% for the Group 2, p = 0.000045). (Table 1)

**Figure 2:**
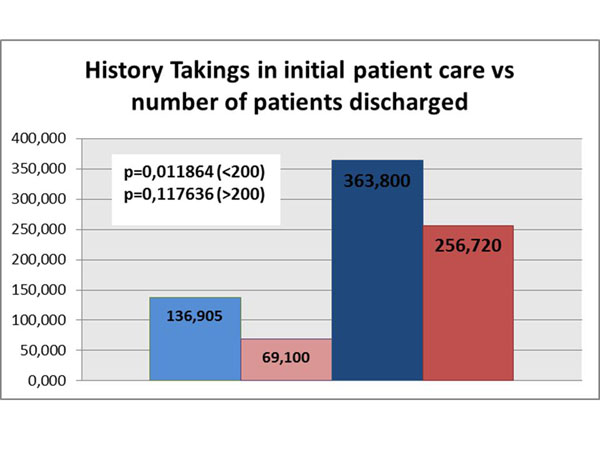
**Number histories takings in initial patient care vs number of patients discharged**. Rose: Group 1 patient discharged. Light-Blue: Group 1 histories taken. Red: Group 2 patient discharged. Dark-Blue: Group 2 histories taken.

In order to achieve the clerkship objectives, it is important for the students to participate in all parts of patient care, from the patient admission in the ED to the management (discharge, admission to hospital floor, admission to ICU, admission to mini-unit, etc). However, these objectives are not required. Consequently, the study found that while students from Group 1 aided in discharging the patient, 69.1 times on average, Group 2 performed the same task 256.7 times ( a 271.5% increase for Group 2). In addition, correlation with the numbers of histories taken revealed that in Group 1, 49.6% of patients whose history had been taken were not followed up and discharged by the same student. In comparison to Group 2, this percentage decreases to 29.4% (p = 0.011 for Group 1, p = 0.117 for Group 2). Concerning the number of supervised prescriptions, Group 1 students wrote 56.7 prescriptions at discharge, and Group 2 students wrote 232.4 (309.9% more). (Figure 2)

**Figure 3:**
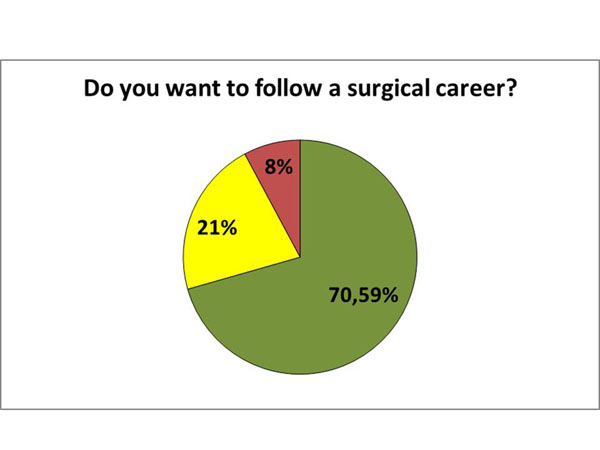
Percentage of Students that want to follow a surgical career. Yellow: No. Red: Not Answered. Green: Yes.

**Figure 4:**
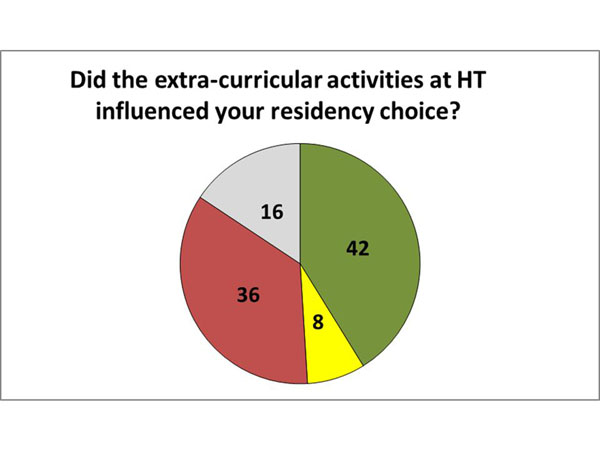
**The supervised extra-curricular practical activity influence in their Decision**. Green: Yes, Positive. Yellow: Yes, Negative. Red: No. Grey: Not Answered

Finally, students were asked about their intention to pursue a surgical career. The vast majority of students (70.6%) said they want to be surgeons, 21.6% said they have no interest in surgical careers and the remainder (7.8%) did not answer the question. Also, when asked if the participation in this clerkship influenced their choice, we found that in 41.6% of cases, the clerkship had a positive influence, 7.8% had a negative influence and 35.3% reported it did not influence their decision. However, 15.7% declined to answer the question. (Figure 3) (Figure 4)

## Discussion

There are only a few studies in the field of extra-curricular academic learning activities, probably because of the methodological difficulty in this form of study. [7,8] However, the rising incidence of students seeking alternative ways to learn medicine and increase their knowledge and skills makes it an extremely important issue that needs to be addressed.

Data collected reflects a major difference between the two groups of students. There are many reasons why students withdraw from the clerkship before they accomplish enough hours to fulfill the requirements for a proper certificate. Personal issues, excessive workload, the increasing service demand, night shifts, lack of sympathy of the health care providers may all be suggested as causes for abandoning the clerkship. However, those students who go on to complete the 200 hours appear to be well ahead in knowledge, skill and medical maturity.

Students in Group 2 outperformed students in Group 1 countless times over. This observation can be explained by the greater length of stay in the clerkship, so that the student is able to repeat over and over again whatever is needed to get used to it.

Furthermore, Group 2 requested 119.7% more radiographs than the Group 1 did. This number seems to be higher only because of their greater length of stay in the service, probably having no direct connection with the quality of their request or need for patient evaluation. However, when we interpret this with the number of supervised evaluations and follow up of the radiographs that the students performed, Group 2 did it almost four times more than Group 1 (273.8%). This seems to be related to better learning, and may even be a sign of maturity, as students begin to understand their own educational process. It is necessary for them to help in every steps of patient care to get the best picture in a better perspective of the entire process.

Also, the number of immobilization and sutures are directly proportional to the student’s number of hours in the clerkship. Although it can be assumed that the more a procedure is performed the better the student’s skill is, it has been proved that self-evaluation is not reliable as a good method to assess abilities [7]. Rather, objective assessment should be applied.

Considering all fields, Group 2 made significantly more of the following procedures: 229% more plaster immobilizations, 211.2% more non-plaster immobilizations, 183.7% more single stitch sutures, 131% more Donatti stitch sutures and 650.2% more Resuscitation Room patient care, which reflect their experience and knowledge for future practice.

We can also observe that students in Group 2 discharged 187.6 times more patients than the ones in Group 2, what can also be explained by more hours in the clerkship. However, if we correlate the number of history taking with the number discharge orientation given to patients, we will find that in Group 2 only 29.4% of patients did not receive proper instructions and follow up, whereas this number rises to 49.6% in the Group 1.

Considering the possible harmful effects of having medical students working in an emergency department alone, all activities developed must be under supervision, what help their practical training process that will never be achieved only by books.[5,9] Nevertheless, replacing curricular activities by extra-curricular ones shall be always discouraged. Not only but also, many Universities unfortunately do not offer a good enough plan of activities for their medical students, making regular lectures not a priority in their schedules (an issue that shall be addressed in a different paper).

Despite the better quality of medical care that can be offered by dedicated doctors compared to medical students, in Brazilian busy public emergency rooms most of the time it is impossible to dedicate the appropriate time on consultations to each patient (what may be the reality in most of the countries worldwide). Then, a team of committed medical students can be extremely helpful on patient care.

Even being non-licensed not-fully-trained, if properly supervised, they can play an important role in this environment. Tutors must be always aware of eventual medical errors that if not promptly approached will be under their legal responsibility as well as a threat to patients’ safety.

Since it is a surgical clerkship, it is expected that the vast majority of students aim to follow a surgical career beforehand (70.6%). However, data concerning the influence of the extra-curricular activity in their decision should be analyzed carefully. Most of the students that do not have interest on a surgical career and find the practical activities a bad influence for them may abandon it before its completion (500 hours), or even before 200 hours, and would not participate in the present study.

## Conclusions

Our data suggests that 200 hours seems to be a suitable threshold in medical students’ learning surgical manual dexterity in an Emergency Department clerkship, even in the absence of objective parameters to further evaluate this theory. Last but not least, maturity and quality of medical care significantly improved proportional to the number of hours served in the ED clerkship. Therefore the practice of leaving before 200 hours should be actively discouraged.

Further comparative studies with objective criteria to evaluate students and residents’ manual dexterity and their own perception of their abilities should be performed in order to assess our initial findings.

## Competing interests

No competing interests declared.

## Authors' contributions

PGTAR conceived the study idea, conducted the study design, writing and applying questionnaires, data analysis and contributed writing the manuscript and translation into English. GCO contributed applying questionnaires, helped with the data analysis and to write the manuscript. ACO participated applying questionnaires, data analysis and writing the manuscript. HS participated in the design of the study, performed the data analysis and discussion, and writing the manuscript and translation into English. AN participated in the design of the study, performed the data analysis, coordination and helped to draft the manuscript. FDST participated in the design of the study, performed the data analysis, coordination and helped to draft the manuscript. All authors read and approved the final manuscript.
